# Cost-effectiveness analysis of neoadjuvant versus adjuvant chemotherapy for cT2-4N0-1 non-small cell lung cancer patients during initial treatment phase

**DOI:** 10.1186/s12962-021-00280-w

**Published:** 2021-07-19

**Authors:** Dongdong Wu, Juan Li, Yubo Wang, Hao Huang, Chunji Huang

**Affiliations:** 1 Department of Information, Daping Hospital, Army Medical University, Chongqing, China; 2 Department of Oncology, Daping Hospital, Army Medical University, Chongqing, China; 3 Respiratory Department, Daping Hospital, Army Medical University, Chongqing, China; 4 Army Medical University, Gaotan Rock, Shapingba District, Chongqing, 400038 China

**Keywords:** Cost-effectiveness, Neoadjuvant chemotherapy, Adjuvant chemotherapy, Non-small cell lung cancer

## Abstract

**Objective:**

The choice between neoadjuvant chemotherapy (NAC) and adjuvant chemotherapy (AC) remains controversial in the treatment of non-small cell lung cancer (NSCLC). There is no significant difference in NAC and AC’s effectiveness. We investigate the cost-effectiveness of NAC versus AC for NSCLC.

**Method:**

A decision tree model was designed from a payer perspective to compare NAC and AC treatments for NSCLC patients. Parameters included overall survival (OS), surgical complications, chemotherapy adverse events (AEs), treatment initiation probability, treatment time frame, treatment cost, and quality of life (QOL). Sensitivity analyses were performed to characterize model uncertainty in the base cases.

**Result:**

AC treatment strategy produced a cost saving of ¥3064.90 and incremental quality-adjusted life-years (QALY) of 0.10 years per patient with the same OS. NAC would be cost-effective at a ¥35,446/QALY threshold if the median OS of NAC were 2.3 months more than AC. The model was robust enough to handle variations to all input parameters except OS. In the probability sensitivity analysis, AC remained dominant in 54.4% of simulations.

**Conclusion:**

The model cost-effectiveness analysis indicates that with operable NSCLC, AC treatment is more cost-effective to NAC. If NAC provides a longer survival advantage, this treatment strategy may be cost-effective. The OS is the main factor that influences cost-effectiveness and should be considered in therapeutic regimes.

**Supplementary Information:**

The online version contains supplementary material available at 10.1186/s12962-021-00280-w.

## Introduction

Non-small cell lung cancer (NSCLC) is a frequent malignancy and the most common cause of cancer-related deaths among males and females globally, resulting in a large social and economic burden [1]. The National Comprehensive Cancer Network (NCCN) guidelines recommend surgery followed by adjuvant chemotherapy (AC) for cT2-4N0-1 NSCLC with a supplementary instruction that neoadjuvant chemotherapy (NAC) followed by surgery should also be considered for these patients [2]. The use of AC for resectable NSCLC has been well established by several randomized trials and meta-analysis, which demonstrated clear survival benefits over surgery alone [3–6].

Evidence of the benefits of NAC is not very strong despite similar overall survival (OS) and disease-free survival (DFS). The original purpose of administering chemotherapy before surgery included: improving operability by reducing tumor tissue size, increasing the likelihood of administering the maximum planned dose of chemotherapy, and reducing the likelihood of micro-metastasis and incomplete resection (R1/R2) [7, 8]. At the same time, there were several potential risks of NAC including delayed operation, increased postoperative complication for the postoperative toxicity, and making the tumor unresectable [9]. Thus, there is widespread debate in the use of NAC and AC with NSCLC patients.

The comparative effectiveness of NAC versus AC in terms of DFS and OS remains controversial. A study by Brandt et al. evaluated whether the treatment strategy of NAC or AC was better for cT2-4N0-1 NSCLC patients through a propensity score match analysis [10]. They analyzed 92 matched-pair patients and ultimately demonstrated that there was no significant difference in DFS and OS between treatment cohorts. Previous investigators have tried to answer this question as well. The NATCH trial recruited 624 patients with stage I–IIIA, N0–N1 NSCLC to compare the effect of three therapeutic strategies (NAC, AC, and surgery alone) [3]. The three arms of the trial found that there was no significant difference in DFS and OS between those treatments. The open label randomized trial by Westeel et al. showed the same conclusion in early stage NSCLC patients [11]. Coincidentally, the meta-analysis of trials also did not demonstrate differences in OS and DFS between NAC and AC [12, 13].

Although they showed similar clinical outcomes, most of the previous literature supports the use of AC over NAC. However, some theoretical differences have not been specifically addressed or adequately studied. Firstly, administration of NAC could have the potential to reduce tumor size before surgery and increase the complete resection rate [9]. Secondly, patients who receive NAC might have higher surgery complications and mortality rates, due to the preoperative chemotherapy toxicity [7]. Thirdly, patients who receive NAC may have better chemotherapy tolerance than the patients who underwent AC alone [3, 10].

These theoretical differences of AC and NAC may impact the cost associated with caring for NSCLC patients. Furthermore, under the circumstances, there was no robust evidence on the outcomes of NAC versus AC in terms of OS and DFS. Cost-effectiveness analyses may contribute to decision-making among NSCLC patients for whom the optimal therapeutic regimen is unclear. Cost-effectiveness research comparing the NAC and AC treatment protocols in lung cancer has been absent in past studies. Previous cost-effectiveness studies about NAC versus AC, focused only on ovarian cancer and head and neck cancer [14–20]. Therefore, in this study, we compared the cost-effectiveness of AC and NAC treatment strategies in cases of NSCLC, through a decision tree-modeled cost-effectiveness analysis from the perspective of the payer.

## Method

### Model structure

We created a decision tree model using the software package Tree-Age pro 2011 to compare the health and economic impact of NAC and AC for cT2-4N0-1 NSCLC patients from the payer’s perspective. Costs were measured using a China Medicare care perspective, and outcome of patients included OS, quality-adjusted life-years (QALYs), health utilities value and treatment time. The utility of disease was calculated according to published utilities and the study of Brandt et al. [10] informed the outcome.

**Fig. 1:**
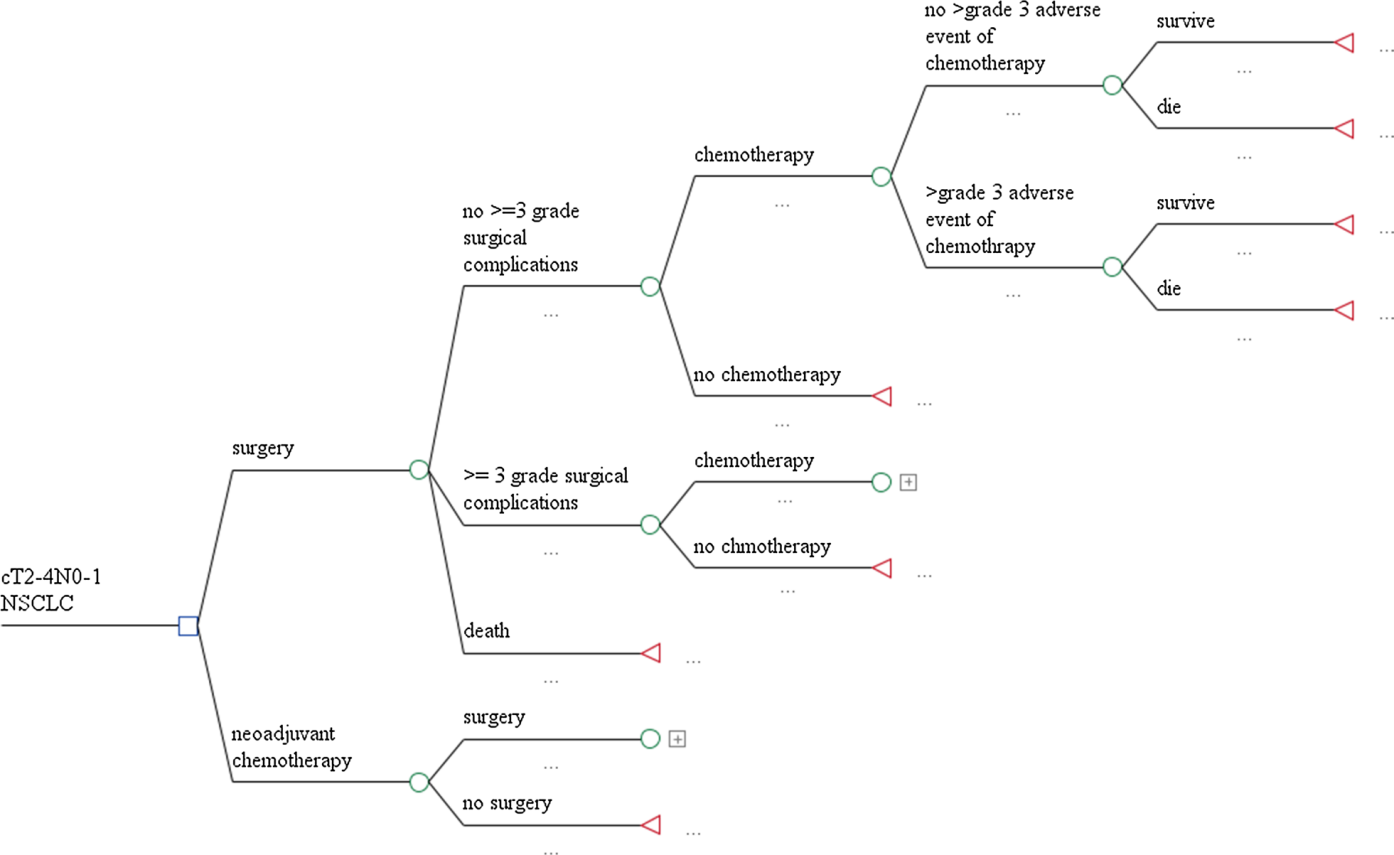
Decision tree model structure comparing NAC to AC for cT2-4N0-1 NSCLC patients

In the model, patients received either surgery followed by four rounds of AC, or two rounds of chemotherapies followed by lung surgery and an additional two rounds of chemotherapies. We assumed that the chemotherapy regimen was intravenous paclitaxel/carboplatin since a previous study showed no significant association of chemotherapy regimens for NAC and AC and no significant difference in the survival for different chemotherapy regimens [10]. For every set of chemotherapy cycle, patients could experience grade 3 or 4 chemotherapy-related adverse events (AEs). Additionally, during surgery (both NAC and AC), patients could also experience surgery complications. If patients experienced AEs or complications from chemotherapy or surgery respectively, they could either recover or die from the event (Fig. 1). The goal of our study is to compare the cost-effectiveness in the initial stage and the treatment stage for cT2-4N0-1 NSCLC patients. Costs and prognosis for patients treated with NAC and AC during the progressive stages were not included in the model.

### Effectiveness and quality of life

The study by Brandt et al. showed no significant difference in median OS between NAC and AC. Therefore, we assumed that the OS (9.1 years) and quality of life (QOL) during the progression-free survival stage were equal. The parameters such as probabilities of AE and complications are also based on the published results of this study (Table 1). Data for postoperative deaths could not be sourced for this study, as such, 30-day mortality and 90-day mortality information was integrated into postoperative complication mortality. Patients treated with NAC all received lung cancer surgery, and patients treated with surgery as the initial therapy, all received AC in this study. For chemotherapy tolerance, we take the proportion of grade 3 and 4 AE as the input parameter. There were no deaths for chemotherapy and AEs.

**Table 1:** Base-case probabilities and cost estimates in the decision tree analysis

Event	Probability		Source
	NAC	AC	
Receiving surgery	1	1	Brandt et al. [10]
Receiving chemotherapy	1	1	
Postoperative complication			
No ≥ 3 grade complication	0.82	0.91	
≥ 3 grade complication	0.14	0.07	
Death	0.04	0.02	
Adverse event of chemotherapy (> 3 grade)	0.15	0.38	
OS	9.1 years	9.1 years	

QOL was estimated using standard health utility weights. The utility weights of stable disease and progressive-free disease were calculated according to published studies [21, 22]. Since there is a shortage of QOL studies for lung cancer surgery complications, we used the utility value of pneumothorax from the study by Handorf et al. [22] for grade 3 and 4 complication utility weights. The average utility weight of every treatment procedure is shown in Table 2, and the time frames of each treatment are based on the study of Dendulur et al. [23] and Lugg et al. [24]. In the model, the treatment of patients with grade 3 or 4 AEs will add 0.73 months to the basic time of surgery and chemotherapy, while treatment of those with grade 3 or 4 complication will add 0.5 months. The utility weight of patients with grade 3 or 4 AEs and complications were 0.45. The utility weight 6 months after the initial treatment remained unchanged.

**Table 2:** Utility weights used for quality-adjusted life-years in the decision tree analysis

	Utility weight	Time frame (months)	Source
Surgery + chemotherapy	0.81	4.87	Dendulur et al. [22] Grutters et al. [23]
≥ 3 grade AE	0.45	0.73	
≥ 3 grade complication	0.63	0.50	Handorf et al. [25] Lugg et al. [26]
Surgery alone	0.77	0.53	Dendulur et al. [22] Grutters et al. [23]
Chemotherapy alone	1.00	0.21	
Death	0	–	–

### Cost

Since the study focused on treatment strategies in the initial treatment phase, costs associated with the treatment of recurrence and rehabilitation were assumed to be equivalent for NAC and AC. Further, we assumed that the two treatment strategies incurred the same costs for biochemical testing, pathological examination, venipuncture related protocols and diagnosis since there are no differences in NAC and AC treatments according to the NCCN guideline. Therefore, cost calculations were done only for medical related items that may have huge differences in NAC and AC treatments.

These costs included those associated with (medical) caring for NSCLC patients including the cost of the surgery procedure, major late complications, chemotherapy administration, and chemotherapy AEs (Table 3). The final cost of the surgery (Additional file 1: Table S1) was calculated as the sum of base surgical procedure costs, additional surgical procedure costs and hospitalization costs (including the cost of surgery complication). The surgery (pneumonectomy, bi-lobectomy, lobectomy, or segmentectomy) costs were constructed from medical program fee schedules (MPFS) [25]. The chemotherapy costs measured the cost of main chemotherapy drugs (paclitaxel and carboplatin). Based on recommendations from the current guidelines, one cycle chemotherapy dosing was calculated as 200 mg/m^2^ for paclitaxel and an area under the curve of six for carboplatin [2]. The imaging examination of the chest, epigastrium, head, and whole-body bone were recommended in diagnosing and pre-operation for NAC, and only in diagnosing for AC patients; imaging examination of chest was recommended in cessation of chemotherapy for both NAC and AC patients [2]. The costs of imaging examinations were calculated as enhanced CT costs, according to the NCCN guidelines.

**Table 3:** Cost estimates for cost-effectiveness analysis of NAC and AC

Event	NAC	AC	Source
Surgery^a^	2351.96	2367.82	MPFS [30]
Additional surgical procedures^a^	1854.46	2513.65	
Hospitalization			
With ≥ 3 complication	96,462	96,462	Xin et al. [27]
Without ≥ 3 complication	66,800	66,800	
Chemotherapy (4 cycles)	30,946.5	30,946.5	Local charge [30]
Imaging	6036	3568	Local charge [30]
≥ 3 grade chemotherapy adverse event treatment^b^	3679.8	3679.8	CTCAE [28] CSCO [29]

^a^Cost was calculated as the weighted average of results reported in Brandt et al. [10]; see Additional file 1: Table S1 for individual procedures and probabilities

^b^Cost was calculated as the weighted average of results reported in NATCH [3]; see Additional file 2: Table S2 for individual procedures and probabilities

The major high-grade chemotherapy AEs were neutropenia, thrombocytopenia, anemia, nausea and vomiting, febrile neutropenia, diarrhea, hyperglycemia, arthralgias, myalgias, fatigue, sensory neuropathy, and allergic reactions [3]. The classification of the AE grade followed that of the Common Terminology Criteria for Adverse Events (CTCAE) Version 5.0 [26] and Guideline of Chinese Society Oncology (CSCO) Primary Lung Cancer [27]. The costs of AEs were estimated by the core medical care components, empirical data, and expert opinion on implementation strategies.

All cost estimates were calculated in 2020 RMB with the medical care components of Consumer Price Index from the price dataset in the Shanghai Price Control Administration [25].

### Sensitivity analyses

Several one-way sensitivity analyses were performed to address the uncertainty of costs and outcomes in our model. The parameters listed in Tables 1, 2, 3 were varied in specified ranges of NAC and AC. In the base-case analysis, a variance of ± 20% to the original values was set as the upper and lower limits.

Probabilistic sensitivity analysis (PSA) was conducted to assess the combined effect of multiple parameter uncertainty on the increment cost-effectiveness ratio (ICER). Factors that varied the sensitivity analyses included OS, base-case event probabilities, costs, utility weights and treatment time frames with distributions selected based on published literature [28]. Cost and treatment time obeyed gamma distribution, with a standard error of 10% of the original value. Utilities distribution reflected a triangular distribution, and base-case utility weights were set as the most likely value with minimum and maximum values of ± 0.05. Event probability distribution was beta distribution, with alpha and beta parameters calculated using the event’s incidence in the study by Brandt et al. [10]. The PSA was performed with 10,000 Monte Carlo simulations across all distributions.

## Result

### Base-case

AC is the dominant strategy in this model with better QOL and lower cost (ICER: ¥− 31,615.2/QALY). Using the base-case probabilities and cost, the AC cost is ¥108,999.73 per patient, which is ¥3064.90 lower than the NAC cost (¥112,064.63 per patient) in the initial treatment phase. The average QALY is 8.66 years for AC and 8.56 for NAC.

### Sensitivity analyses

**Fig. 2:**
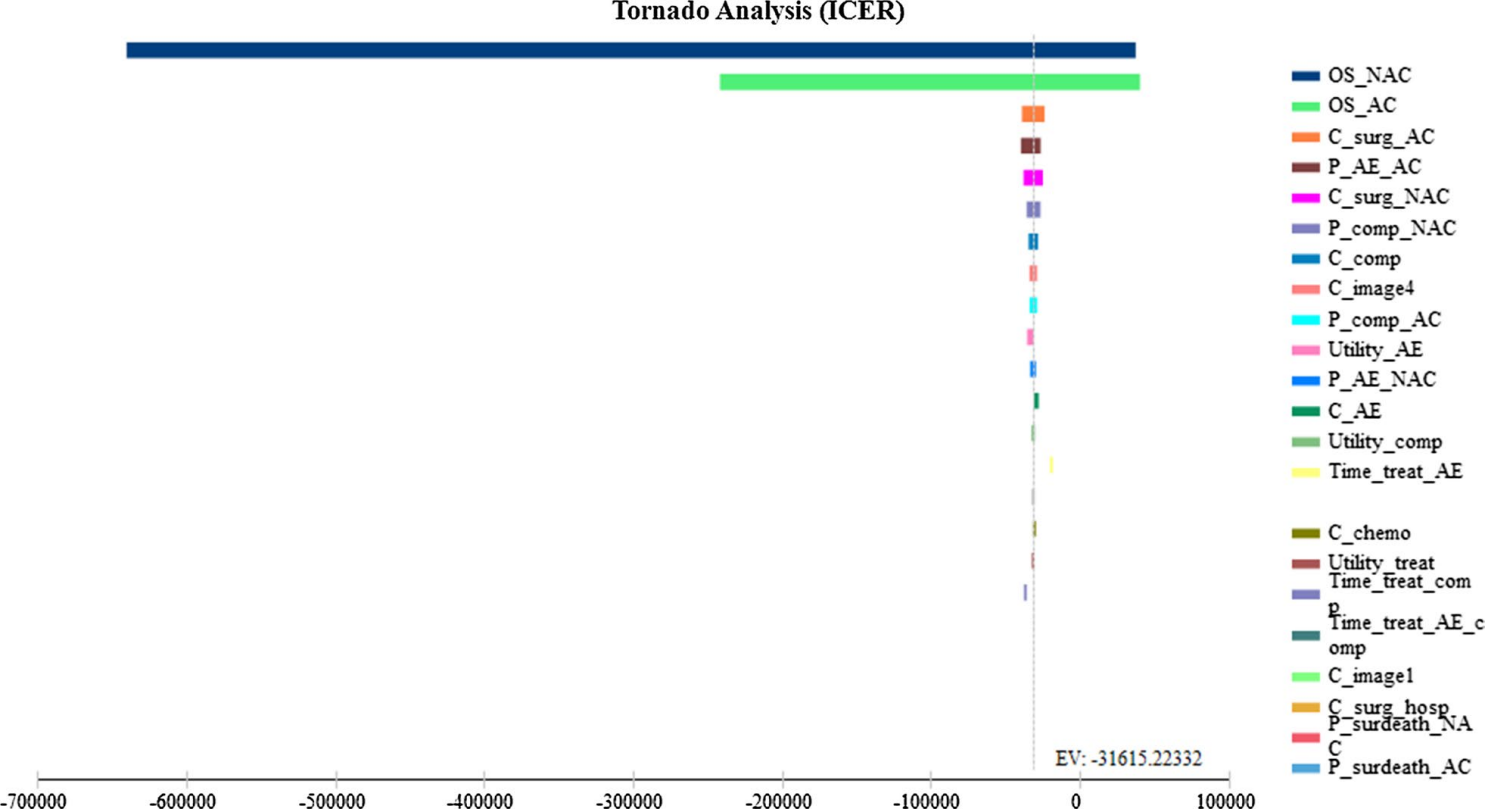
Tornado diagram showing the impact of each varying parameter on the ICER in the model

**Fig. 3:**
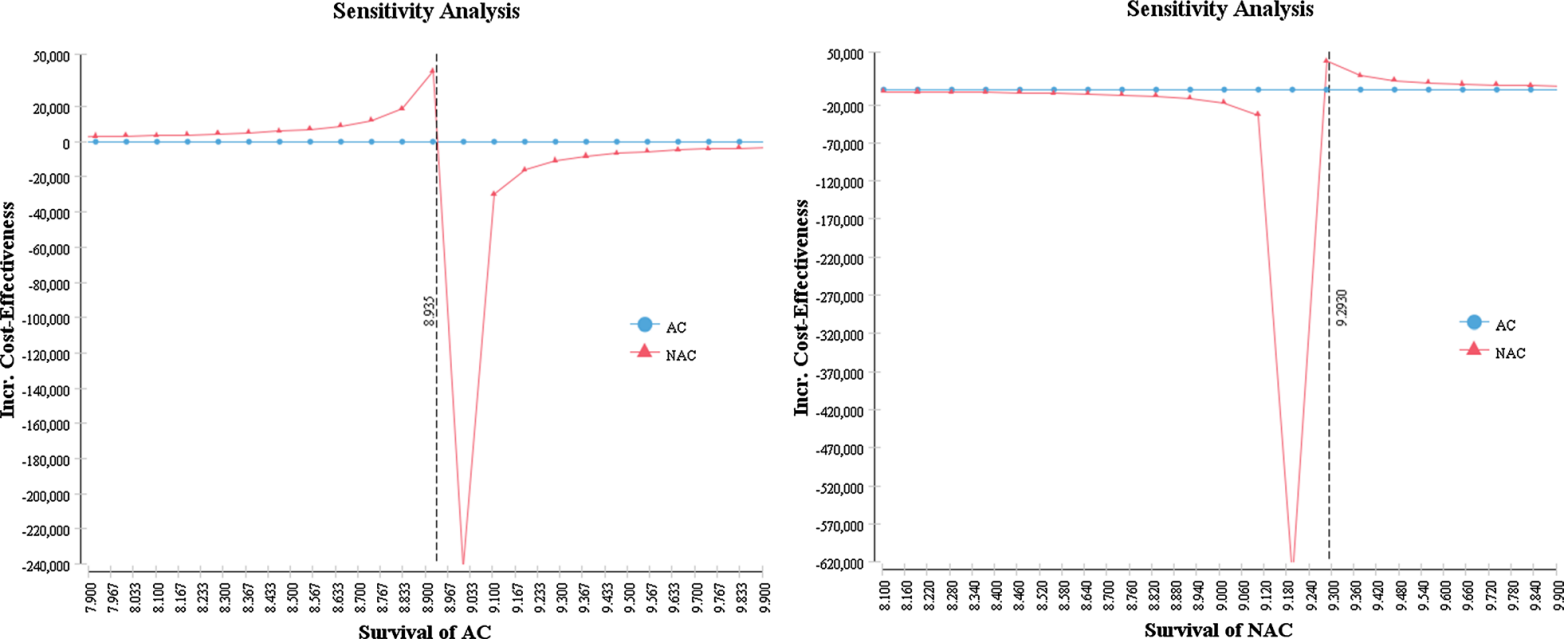
One-way sensitivity analysis for the impact of the overall survival on the incremental cost-effectiveness

We performed a series of one-way sensitivity analyses to discover the effects of the different parameter’s uncertainty on the ICER. The Tornado diagram shows the effect of those parameters that led to a change in the ICER from large to small (Fig. 2). In our model, the ICER was most sensitive to variations in OS of NAC and AC when the median OS of NAC is 0.19 years more than AC; the NAC will be cost-effective at the ¥35,446 threshold (Fig. 3). The sensitivity analyses suggest that our model was robust enough for the uncertainty surrounding the parameters of cost, base-case probabilities, treatment time, and utility weight.

**Fig. 4:**
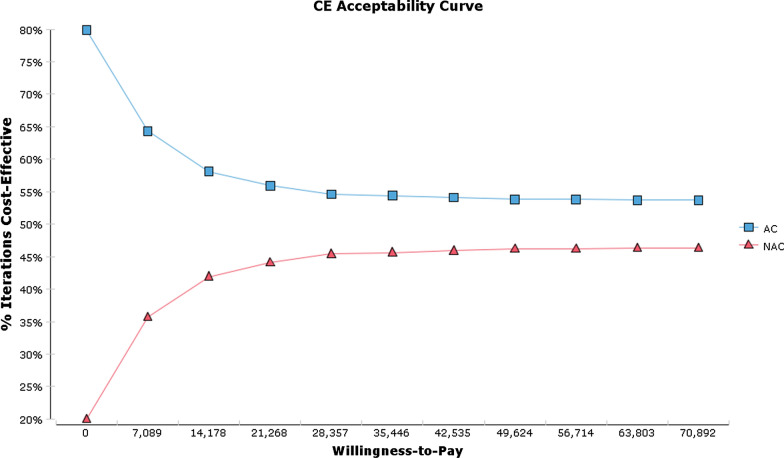
Cost-effectiveness acceptability curve from PSA

In PSA, AC was the dominant strategy in 68.9% of 10,000 Monte Carlo simulations at the threshold of ¥35,446 per QALY; there is a 54.4% probability that the AC strategy is cost-effective. Figure 4 shows the corresponding cost-effectiveness acceptability curve for NAC and AC. Therefore, the statement about basic simulation regarding incremental costs, effectiveness and the ICER, is stable.

## Discussion

The cost comparison between NAC and AC for lung cancer patients has not been extensively studied. We began our study searching the PubMed database to identify studies published before January 2020 that analyzed the cost-effectiveness of NAC and AC in NSCLC using the following search terms: adjuvant chemotherapy and neoadjuvant chemotherapy, cost-effectiveness; cost-effectiveness, adjuvant chemotherapy, neoadjuvant chemotherapy and lung cancer; cost-effectiveness, preoperative, postoperative, chemotherapy and lung cancer. Seven studies evaluated the cost-effectiveness of NAC and AC; however, none assessed the cost-effectiveness specific to lung cancer. Of the seven cases mentioned, six were studies related to ovarian cancer and one related to head and neck cancer [14–20].

Among these studies, head and neck cancer revealed that NAC is more cost-effective than AC [20]. Four of the studies [14–17] related to ovarian cancer showed similar results and other two studies [18, 19] indicated AC as the dominant strategy. Findings from previous research studies stated that the therapeutic regimen is more cost-effective but these findings are not consistent.

The findings in our study showed that NAC is more cost-effective than AC, with a cost saving of ¥3064.90 and a QALY increment of 0.10 years per patient. In contrast to previous studies, the input parameters in our model included the cost of chemotherapy AEs; only one study by Tran et al. [14] explicitly incorporated the chemotherapy AE into their model. One possible explanation for is that there were no significant differences in the chemotherapy-related toxicities for NAC and AC in ovarian cancer and head and neck cancer [15, 29]. For NSCLC patients however, the tolerability of chemotherapy and the ratio of AE are significantly different in NAC and AC as supported by the NATCH three-phase trial [3] and the study by Brant et al. [10]. Nonetheless, the difference in tolerability of chemotherapy and the ratio of AE does not contribute to OS. In addition, the treatment expense of grade 3 and 4 AEs are even higher than the surgery procedure cost [14]. Thus, although the result in our model was not sensitive to the ratio and cost of AEs, we believe the cost comparison between NAC and AC needs to consider the impact of AEs.

The sample population in our study is cT2-4N0-1 NSCLC patients excluding stage IV patients (for whom NCCN guidelines recommend two treatment strategies). The choice of NAC and AC is a tough one in the initial treatment phase. The patients who are less clinically at-risk benefit more from AC, while the stage IV patients are recommended systemic therapy by NCCN and there is robust evidence in support of same [2]. Thus, our study focused on the sample population of patients whose treatment strategies were controversial.

However, most studies compared NAC or AC with the treatment of surgery alone, and estimated the survival benefit; very few studies directly compared the two chemotherapy approaches [7, 8]. The head-to-head comparison of the studies of NATCH and Brandt et al. in light of NAC and AC, showed that there were no statistically significant differences in the OS and DFS. However, the NATCH trial was criticized for being overly optimistic and over representing the study design [7, 8]. The percentage of stage I diseased patients in the cohort who did not benefit from chemotherapy is 75%. In comparison with the meta-analysis [12], the stage I diseased patients in the NAC cohort accounted for nearly 50% of the group. This is the reason base-case probabilities are centered on the study of Brandt et al. in our model.

Furthermore, our study used real-world data. The study generated two groups (92 in NAC and 92 in AC) with comparable characteristics through strict exclusion criteria and propensity score matching analyses to prevent selection bias related to a nonrandomized cohort. The ratio of males and females more closely reflects the real-word population of NSCLC patients who need to receive either NAC or AC.

What is more, the study sample population excluded the patients with microscopic and macroscopic residual disease (R1/R2 resection), which avoids the influence of surgery discrepancy (since the surgery which results in resection to minimal or no gross residual disease may be associated with a long-term survival advantage). The single-center data source reduced the effectiveness of surgery.

There are some limitations to our model. As with all cost-effectiveness analyses, assumptions in clinical base-cases, cost and QOL are important to the projected outcomes determined by the model. Consequently, one-way and probability sensitivity analyses were performed to test our assumptions. The sensitivity analyses showed that our model was robust enough to handle to the variation of cost, QOL, ratio of complication and AEs. However, the variation of OS would change the conclusion of the cost-effectiveness analysis in our model.

The median OS is the most sensitive parameter in our cost-effectiveness analysis model. The studies of Brandt et al., the NATCH trial and Tim et al. all showed that the median OS of NAC and AC have no significant differences [3, 10, 12, 13]. In fact, the difference (< 0.19 years) of the median of NAC and AC (9.22 vs. 8.98 year in Brandt et al.) is enough to change the conclusion of our model. If 9.22 and 8.98 years as the OS of NAC and AC in our model are used, then NAC is more cost-effective with the ICER of ¥22,560/QALY. Given the concern of survival in lung cancer treatment for NSCLC patients, it is important to evaluate sensitivity of OS in cost-effectiveness analysis.

Simultaneously, there are several assumptions in the cost. To make the model clear and accurate, our cost measures were intentionally confined to the associated costs of the initial treatment phase. This was also based on the assumption that there were no significant differences between treatment and ongoing care in the NAC and AC groups beyond the initial recovery period. However, if long-term surgery complication or chemotherapy AEs affected one group and increased the follow-up medical treatment, the difference of NAC and AC cost may be improperly over or underestimated. Furthermore, one patient may not have once AE in the chemotherapy treatment.

In addition, probabilities used in estimating surgery complication and postoperative death may be overrated in NAC because patients with comorbidities or more complex diseases may be more likely to receive NAC. Hence, the reason the ratio of related complication in NAC is higher than AC in our model. In the NATCH trial however, the postoperative death of AC is higher than NAC (5% vs. 7.5%) and the ratio of complication in the multicenter randomized controlled trial (RCT) is influenced by the level of the surgery team. For the case of chemotherapy tolerance, our model did not consider the probability of completed chemotherapy (full dose and full cycles). The chemotherapy AEs of NAC and AC had no significate difference (25.4% vs. 27.3%) in the NATCH trial. Thus, the base-case probability may change in the future with more comparative research in the area of NAC and AC.

Currently, no comparative studies have examined QOL in NAC and AC for NSCLC patients [31]. Hence, we assumed that the health utility weight of NAC and AC is the same at the various treatment stages. We also used health utility weights from previously published literature with NSCLC treatment phase whenever possible. There is also a difference in psychological effects after NAC and AC.

## Conclusion

Despite the higher levels of chemotherapy tolerance and the same survival rate in NAC and AC, AC has a favorable cost-effectiveness profile in the NSCLC initial treatment phase. The cost-effectiveness analysis is sensitive to survival at classic willingness-to-pay thresholds. Based on the findings in this study, NAC as a first choice treatment in cT2-4N0-1 NSCLC patients is not supported. To better assess the relative merits of these therapeutic regimens, attention should be given to OS and DFS as well as the QOL and cost-effectiveness, especially when the number of lung cancer patients and treatment burden increases. In view of the insufficiency in head-to-head trials and clinically enriched datasets, the cost-effectiveness analysis of NAC and AC can benefit from more cohort studies.

## Supplementary Information

**Additional file 1: Table S1.** Cost estimates of base surgery and additional surgery procedures.

**Additional file 2: Table S2.** Cost estimates of chemotherapy adverse events.

## Data Availability

The data and material that support the findings of this study are available within the article or its Additional files.

## Code availability

The study was performed by software package Tree-Age pro 2011. The TreeAge Pro Trex files are available from the corresponding author on reasonable request.
