# Supplementary material for: Cost-effectiveness analysis of neoadjuvant versus adjuvant chemotherapy for cT2-4N0-1 non-small cell lung cancer patients during initial treatment phase
Source: Cost Eff Resour Alloc. 2021 Jul 19;19:44. doi: 10.1186/s12962-021-00280-w (PMC8287679; doi:10.1186/s12962-021-00280-w)
Supplement: Supplementary file 1 — Additional file 1: Table S1. Cost estimates of base surgery and additional surgery procedures. [file 12962_2021_280_MOESM1_ESM.docx]

Table S1 Cost estimates of base surgery and additional surgery procedures

| Event | probability | | Cost | Source |
| --- | --- | --- | --- | --- |
|  | NAC | AC |  |  |
| Pneumonectomy | 10.87% | 11.96% | 2821.5 | MPFS[29]  Brandt et al.[10] |
| Bilobectomy | 5.43% | 6.52% | 3240.5 |  |
| Lobectomy | 80.43% | 79.35% | 2227.5 |  |
| Segmentectomy | 3.26% | 2.17% | 2376 |  |
| Wedge | 0.00% | 0.00% | 1633.5 |  |
| Lymphadenectomy | 54.35% | 67.39% | 2640 |  |
| pleurectomy | 21.74% | 38.04% | 1930.5 |  |
