# Supplementary material for: Cost-effectiveness analysis of neoadjuvant versus adjuvant chemotherapy for cT2-4N0-1 non-small cell lung cancer patients during initial treatment phase
Source: Cost Eff Resour Alloc. 2021 Jul 19;19:44. doi: 10.1186/s12962-021-00280-w (PMC8287679; doi:10.1186/s12962-021-00280-w)
Supplement: Supplementary file 2 — Additional file 2: Table S2. Cost estimates of chemotherapy adverse events. [file 12962_2021_280_MOESM2_ESM.docx]

Table S2 Cost estimates of chemotherapy adverse events

| 3 and 4 grade AE | No. | Cost (RMB) | Source |
| --- | --- | --- | --- |
| neutropenia | 34 | 3542 | NATCH [3]  CTCAE [27]  CSCO[28]  MPFS[29]  Expert Opinion |
| thrombocytopenia | 4 | 11907.02 |  |
| anemia | 3 | 7431.61 |  |
| nausea & vomiting | 8 | 1112 |  |
| febrile neutropenia | 5 | 3542 |  |
| diarrhea | 6 | 7810.94 |  |
| hyperglycemia | 8 | 1179.76 |  |
| Arthralgias | 6 | 2164.4 |  |
| myalgias | 3 | 2164.4 |  |
| fatigue | 8 | 5012.2 |  |
| sensory neuopathy | 4 | 395.3 |  |
| allergic reaction | 2 | 223 |  |
| Average cost | 3679.8 | |  |
